# Survey on Revocation in Ciphertext-Policy Attribute-Based Encryption

**DOI:** 10.3390/s19071695

**Published:** 2019-04-09

**Authors:** Ruqayah R. Al-Dahhan, Qi Shi, Gyu Myoung Lee, Kashif Kifayat

**Affiliations:** 1 College of Computer and Information Technology, University of Anbar, Al-Anbar 31001, Iraq; 2 Department of Computer Science, Liverpool John Moores University, Byrom Street, Liverpool L3 3AF, UK; G.M.Lee@ljmu.ac.uk; 3 Department of Computer Science and Engineering, Air University, Islamabad 44000, Pakistan; kashif.kifayat@mail.au.edu.pk

**Keywords:** cloud computing, internet of things, Ciphertext-Policy Attribute-Based encryption, multiauthority schemes, single authority schemes, revocation.

## Abstract

Recently, using advanced cryptographic techniques to process, store, and share data securely in an untrusted cloud environment has drawn widespread attention from academic researchers. In particular, Ciphertext-Policy Attribute-Based Encryption (CP-ABE) is a promising, advanced type of encryption technique that resolves an open challenge to regulate fine-grained access control of sensitive data according to attributes, particularly for Internet of Things (IoT) applications. However, although this technique provides several critical functions such as data confidentiality and expressiveness, it faces some hurdles including revocation issues and lack of managing a wide range of attributes. These two issues have been highlighted by many existing studies due to their complexity which is hard to address without high computational cost affecting the resource-limited IoT devices. In this paper, unlike other survey papers, existing single and multiauthority CP-ABE schemes are reviewed with the main focus on their ability to address the revocation issues, the techniques used to manage the revocation, and comparisons among them according to a number of secure cloud storage criteria. Therefore, this is the first review paper analysing the major issues of CP-ABE in the IoT paradigm and explaining the existing approaches to addressing these issues.

## 1. Introduction

Unlimited cloud storage and data outsourcing services provide data owners and enterprises with capacities for storing and processing a massive amount of data. These high quality services enable easy accessibility, high scalability, and availability [1]. Despite all the advantages, serious concerns about the data security and confidentiality in such a cloud environment have been raised [2]. To preserve data confidentiality, many techniques support searching on the outsourced data stored in the cloud environment. However, these techniques cannot regulate access to specific stored data record or enforce access policies [3].

In an untrusted cloud environment, preserving data confidentiality, making an appropriate decision on data access, and enforcing access policies are core challenges. Therefore, many system models and techniques for access control based on cryptographic operations have been characterized and described by researchers to provide secure and efficient cloud access control. However, cryptographic approaches for lightweight devices have established their own paradigm. Although such techniques enable data sharing among a large number of users, some open issues still need to be addressed, particularly for Internet of Things (IoT) applications. Firstly, access control policies should be expressive enough to incorporate relevant contextual information on the properties, features or characteristics that are associated with users, objects, or the environment (e.g., age, position, time, etc.), reflecting the frequently changing conditions and correlating with ongoing activities in the environment concerned [4].

Secondly, since IoT applications are varying widely, e.g., e-health including patients’ medical records management and remote diagnoses, military systems including soldiers’ data management and monitoring, smart vehicles including traffic jam management, and smart cities, securing such applications by building a collusion resistant system and providing fine-grained access control is a key challenge. The reason for this is that the sensitivity of such application data and the consequences of attacking such applications cause great damage to systems and their users.

Thirdly, the most important issue from the user point of view is how to flexibly join and leave a system with a low computational cost (i.e., efficient user revocation). To address the above challenges, a considerable amount of research has been conducted to develop necessary cryptographic techniques. This paper will critically examine and compare these techniques to demonstrate their effectiveness and efficiency as well as limitations.

In a large-scale distributed environment, particularly the cloud environment, traditional symmetric cryptographic techniques with the same key for both encryption and decryption operations suffer from a key distribution and management problem. On the other hand, traditional asymmetric cryptographic approaches, which utilize a public key for encryption and a private key for decryption, lack computational efficiency. These one-to-one schemes are not desirable to encrypt data and send it to a group of recipients. The reason for this is that a data owner needs to know who the recipients are, their identities, or their public keys before encrypting its data and sending it to the corresponding authorized users separately.

However, to eliminate the enormous computational costs of the traditional cryptographic operations, and to achieve the mentioned requirements (such as preserving data confidentiality, regulating access to stored data in a dynamic environment and using expressive policies), several types of cryptographic access control have been proposed. The most popular types include hierarchical access control and attribute-based access control.

In terms of hierarchical access control, to fulfill confidentiality, the first hierarchical key assignment scheme was introduced by Akl and Taylor [5]. In particular, the data objects need to be shared and are encrypted using one of the symmetric encryption approaches, and users are organized in a partially ordered hierarchy based on their access rights to form security classes. Each class is assigned private and public information for computing the keys of any lower down classes in the hierarchy. However, this system does not support dynamic updates (e.g., insertion, revocation, etc.), in contrast to the highly dynamic large-scale nature of cloud-based storage.

Therefore, a recent study has been proposed [6] to address the limitations of Akl and Taylor [5] by considering different strategies of key assignment. This system deals effectively with the key management problem as well as dynamic updates such as user or class revocation. However, any update occurred needs to reinitialize the private and public parameters for the updated class and any affected lower down classes in the hierarchy. Moreover, the access policies are not expressive enough. These systems are also based on the RSA assumption on the use of a large key size about 1024 bits to be secure and make the discrete logarithm problem hard. For these reasons, the process of key generation and key derivation incurs high computational cost. Therefore, these systems are not suitable for most large-scale applications.

Another piece of work using a hierarchical key assignment scheme is recently proposed [7]. Unlike other relevant schemes that need a single secret and some public information to derive decryption keys, this system does not require publishing any public information for the key derivation operation in the lower down classes because it is based on the concept of chain partitions of the access policy. However, the number of appropriate, additional secrets required to be sent to each authorized user ought to be more than one secret but not more than the number of partitions, leading to the increase in the amount of data sent via the secret channel. In addition, this system does not consider dynamic updates such as deletion, insertion and revocation processes.

On the other hand, expressiveness is fulfilled by introducing attribute-based access control, which grants access privileges to users based on their attributes, e.g., doctor or researcher and health science center, specifies that only doctors or researchers in the Health Science center can access the related data. To perform attribute-based access control while preserving data confidentiality, Attribute-based Encryption (ABE) has been proposed, which is an advanced asymmetric cryptographic technique invented to leverage the merits of the symmetric encryption (e.g., high efficiency) and solve its key management and distribution problems. Such a system is based on elliptic curve cryptography. This makes the discrete logarithm problem hard with a reasonable security level by using a small secret key size (about 160 bits).

Two variants of ABE were discussed in Goyal et al.’s scheme [8]. These variants are ciphertext policy attribute based-encryption (CP-ABE) and key policy attribute-based encryption (KP-ABE). The major difference between them lies in how to associate a secret key and an access policy with relevant data and attributes. In KP-ABE, an access policy is associated with a secret key and a set of attributes are associated with the data encrypted with the key. Conversely, in CP-ABE, each encrypted data item is assigned with a specific access policy and a user’s secret key for the data decryption is assigned with a set of attributes. As a consequence of embedding an access policy into a user’s secret key in KP-ABE, a data owner (who encrypts the data) can only select a set of attributes but will not be able to decide which user can access its ciphertext. To decrypt the ciphertext, a key generator (i.e., an attribute authority), which generates the decryption key for authorized users, will be responsible for granting or denying access to the key [9]. Due to this property, we pay little attention to KP-ABE and focus on CP-ABE in the rest of this paper, as our objective is to give data owners full control of their sensitive data: CP-ABE is more suitable for this purpose.

However, despite the CP-ABE merits, the significant issues that the first proposed CP-ABE schemes [9,10] did not consider are revocation and managing a wide range of attributes. In terms of revocation, in any access control system, this property is essential to emulate the dynamicity of cloud storage. For this reason, many researchers have paid a lot of attention to extend the proposed schemes and resolve the revocation issue with low computational costs to be applicable to IoT devices.

To critically analyze the existing work based on the above stated challenges and requirements, we have to mention that most of the current CP-ABE work achieves data confidentiality and privacy, fine-grained access control, and expressive access structure [10,11]. However, the challenge of performing an efficient revocation process is still open. Therefore, in this review, we put a strong emphasis on the main requirements that have not been efficiently fulfilled yet such as revocation and collusion resistance. To the best of our knowledge, this survey is the first work that examines the types of revocation techniques used in both single and multiauthority schemes built on the most popular variant of ABE, which is CP-ABE. Most of the existing reviews cover the common types of access policies (e.g., monotonic or nonmonotonic access policies) appeared in available literatures. In addition, these reviews are lack of information on research in revocation, although it is the most highlighted, well-known issue.

This review is structured as follows. Section 2 presents the background of elliptic curve cryptography. ABE, its requirements, and the main problems of CP-ABE are defined in Section 3. Section 4 introduces existing single authority and multiauthority CP-ABE schemes. The research challenges and future directions are described in Section 5. Finally, Section 6 concludes the review.

## 2. Elliptic Curve Cryptography

Elliptic curve cryptography (ECC) is a type of public key encryption based on elliptic curve groups over finite fields [12]. An elliptic curve is the set of points with elements of a finite field described by the equation

(1) $$y^{2}\;=\;x^{3}3\;+\;ax+\;b,$$

where a and b are parameters which determine the shape of the curve. In addition, it requires that the discriminant $\Delta \;=4Aa^{3}+27b^{2}$ is nonzero. Equivalently, the polynomial $x^{3}\;+\;ax\;+\;b$ has distinct roots. This ensures that the curve is nonsingular. Moreover, there is a need for a point at infinity φ. So E is the set of

(2) $$E=\;\left\{\left(x,y\right):\;y^{2}\;=\;x^{3}\;+\;ax\;+\;b\right\}\cup \left\{\varphi \right\}$$

Geometry can be used to make the points of an elliptic curve into a group. An elliptic curve group G consists of the elliptic curve points, Q, and a group operation called addition, denoted by ’+’. Furthermore, the point at infinity serves as the identity element, where adding points on an elliptic curve is closure. The addition law on the elliptic curve group has properties that are shown as follows

(a) P+ φ= φ +P=P Ɐ *P* ∈ *E*.
(b) P+ (−P) = φ Ɐ *P* ∈ *E*.
(c) P+ (Q+R) = (P+Q) +R Ɐ *P*, *Q*, *R* ∈ *E*.

The addition operation of elliptic curve groups has the property of being commutative, i.e., for all *P*, *Q* ∈ *G*, then *P* + *Q* = *Q* + *P*. Elliptic curve groups could enable shorter keys, while providing a similar level of security to the conventional multiplicative group of a finite field. Due to the small key sizes of ECC and relatively fast computations, ECC becomes the most popular choice for public key encryption in many applications, especially those which use sensors (e.g., to achieve a 80-bit security level there is a need to use a 1024-bit key in RSA, while only a 160-bit curve in ECC is needed).

The difficult issue that most ECC schemes are based on is the discrete logarithm problem (DLP). This problem can provide a sufficient level of security if the related parameters are chosen properly.

## 3. Attribute-Based Encryption

Attribute-based encryption (ABE) is one of the advanced cryptographic techniques for one-to-many encryption that overcomes the limited functionalities of the traditional public key cryptographic techniques. This scheme was proposed by Sahai and Waters [13] as an application of fuzzy identity-based encryption, which uses human-intelligible identities (such as unique name, IP address, email address, etc.) as public keys, where a data sender directly encrypts its data with the receiver’s identity. Later, Goyal et al. [8] presented a more general construction of ABE, in which attributes have been utilized to issue a public key and to generate a logical expression of these attributes called an access policy. Both the public key and access policy are used for encrypting data. In contrast with the traditional cryptographic systems which encrypt data for one particular user or group that knows the decryption key, there is no more need to share the same private key or store several versions of the ciphertext encrypted with different keys [14]. Moreover, ABE has no restriction on the number of users in the system. Based on these considerations, this scheme has been leveraged to regulate users’ access to cloud data by using attributes as an access policy.

**Figure 1:**
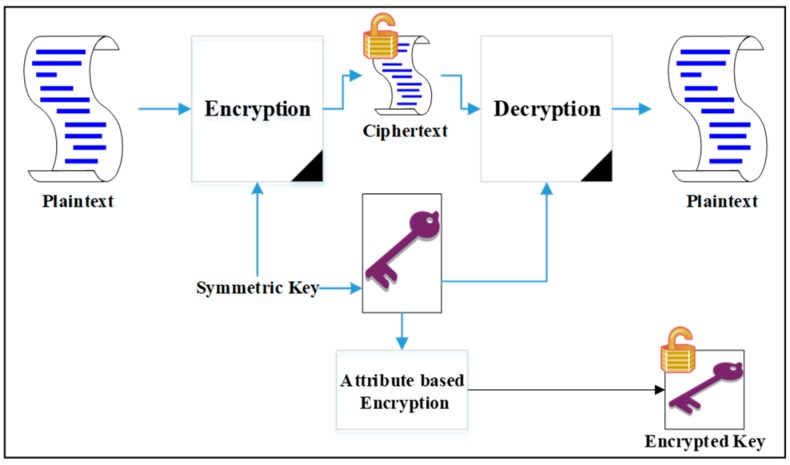
Using ABE to encrypt a symmetric key.

To apply ABE, a data owner encrypts its data using a symmetric encryption algorithm with a symmetric key and then encrypts the key using an ABE scheme with a public key. The encrypted key is distributed to a group of recipients/users as a ciphertext. Each user obtains the private key for the encrypted key decryption from a key generator that calculates the key according to the user’s attributes. In this case, the data owner does not need to know the identities of the legitimate users and their dynamicity. Figure 1 illustrates the above-mentioned operational process.

**Figure 2:**
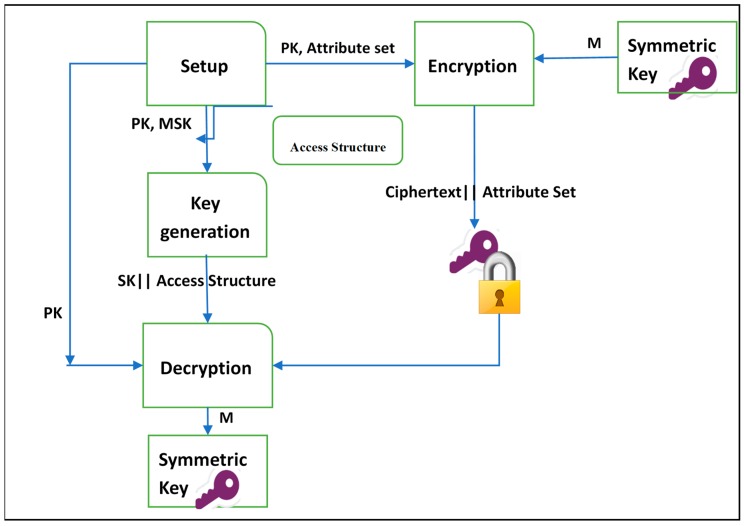
The procedure of key policy attribute-based encryption (KP-ABE).

**Figure 3:**
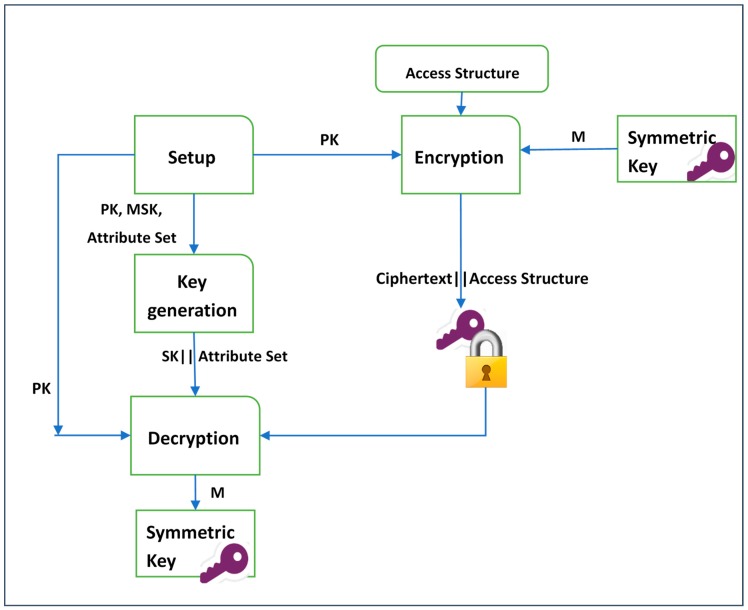
The CP-ABE mechanism.

Applying ABE to the two variants (i.e., CP-ABE and KP-ABE) follows the same procedure as shown in Figure 2 and Figure 3. The main difference is, in KP-ABE, users’ secret keys are issued using an access policy that defines the access privileges of the authorized user, and the symmetric key is encrypted over a set of attributes. However, CP-ABE uses access policies to encrypt data (i.e., symmetric key) and secret keys of the legitimate users are generated over a set of attributes. In addition, some evaluative requirements need to be satisfied by any ABE scheme

1. Data confidentiality and privacy is a set of rules that protects a certain type of information by placing some restrictions on it. It is an essential requirement for cloud storage since the cloud service provider, which stores the data, is normally unauthorized to access its content. Thus, the data accessibility ought to be only for explicitly legitimate users. This is satisfied by using ABE to enable the legitimate data access even when the data encrypter is offline and the privacy preservation of the data users’ identities.
2. Fine-grained access control is a key mechanism that grants different access privileges to different users even if they are in the same group according to their credentials given by the associated system, and flexibly specifies individual users’ access rights.
3. Expressive access structure is important for the access policies specified by a data owner to be expressive in order to realize fine-grained access control. Moreover, ABE is required to support the expressiveness of the policies. This requirement makes the access control similar to a real-life access control.
4. Collusion Resistance. The system has to prevent any collusion attacks from combining their information together to illegitimately gain unauthorized data through collaboration [15]. In the cloud environment, this type of attack can be either a group of misbehaving system users who collude with each other to combine their information and gain higher access rights, or a combination of a cloud server and malicious, revoked users who try to gain the original data.
5. Forward and Backward Secrecy. Forward security means any revoked user ought to be prevented from accessing data and decrypting any new published ciphertext after leaving the system. In terms of backward security, to achieve this type of security, it needs a mechanism in which the ciphertexts which were published previously cannot be decrypted by any user who newly joins the system [16].
6. Revocation. When a user is degraded or leaves the system, its access rights need to be reduced or revoked by the related access control scheme without incurring significant computational cost, respectively. In addition, attribute updating is not a straightforward process in ABE and is hard to address, as updating a single attribute could impact a large number of users accessing the same attribute.
7. Scalability. The performance of the system should not be affected by the increase of system users.
8. Computation overhead is essential to fulfill all the above requirements with minimal computation cost.

Many KP-ABE and CP-ABE schemes were proposed with some notable examples listed in Table 1.

### 3.1. Ciphertext-Policy Attribute-Based Encryption (CP-ABE)

The most popular variant of ABE techniques is CP-ABE. Four entities are responsible for running this scheme. These entities are attribute authority, data owner, data user, and cloud server. The role of the attribute authority is to generate secret keys for users according to their attributes to decrypt data. In addition, it is responsible for generating a public key and a master key. The data owner’s role is to define an access policy that describes who can access to its data as well as encrypting those data under this access policy. Firstly, a data owner uses a symmetric encryption technique (e.g., AES) to encrypt its data. After that, the owner encrypts the symmetric key under its access policy using CP-ABE by selecting a random value as a secret which is shared using the linear secret sharing scheme technique to generate some values associated with each corresponding attribute in the ciphertext according to the owner’s access policy. This policy is determined over a set of attributes by the data owner, and can be demonstrated as a Boolean function with (AND, OR) gates between attributes (e.g., (lecturer AND experience ≥ 2 years) OR Professor). Then the encrypted data is sent to the designated cloud for storage including the data ciphertext, the CP-ABE ciphertext and the access policy. Associating the access policy with the ciphertext means that the ciphertext chooses which key can recover the plaintext, giving the data owner more control of its outsourced data [18]. The eligible users who possess the required attributes in a right combination (i.e., satisfy the access policy) can successfully decrypt the encrypted data. As a result, the main benefit from CP-ABE is that sensitive data can be stored on an untrusted server without performing authentication checks for the data access [19].

A common framework of a CP-ABE scheme includes four algorithms, as demonstrated in Figure 3: Setup, Encryption, Key Generation, and Decryption [10], which are defined below.

- Setup(λ,U)→(MSK,PK)*:* Takes a set of attributes U in the system and an implicit security parameter λ (such as the type of the elliptic curve group used and the base finite field) as inputs to generate a public key *PK* and a master key *MSK* as outputs.
- Encrypt(PK,A,M )→CT*:* Takes as inputs a public key PK, an access structure A, and a message *M* to be encrypted. The output will be a ciphertext *CT*.
- KeyGen(MSK,S)→SK: In this algorithm, a master key *MSK* and a set of attributes *S* are taken as inputs. A user’s secret key *SK* is generated as output.
- Decrypt (CT,SK)→M: This algorithm takes as inputs a user’s secret key *SK* and a ciphertext *CT*. It returns a message *M* when the user’s attributes satisfy the access structure.

There are some appealing merits of the CP-ABE technique over other one-to-one traditional encryption techniques that enable coarse-grained access control. First, CP-ABE is to enable fine-grained access control in an encrypted form. This is desirable for many access control applications that run some cloud services such as storage and sharing services. Secondly, it supports highly expressive policies representing any access structures. Thirdly, it offers a good solution to data confidentiality. As generating a secret key for a user happens only once but it can be used to decrypt all the subsequent ciphertexts, CP-ABE reduces communication overhead [18]. Fourthly, it is collusion-resistant against misbehaving authorized users, which is achieved by associating a random number or polynomial with each attribute of a user’s secret key so that only the attributes with the same random value can be used for decryption, leading to preventing different legitimate users from colluding with each other [20]. Finally, it is possible to integrate CP-ABE with a proxy re-encryption technique in cloud in order to increase security by re-encrypting ciphertexts without disclosing the plaintexts to the cloud [18].

There are some appealing merits of the CP-ABE technique over other one-to-one traditional encryption techniques that enable coarse-grained access control. First, CP-ABE is to enable fine-grained access control in an encrypted form. This is desirable for many access control applications that run some cloud services such as storage and sharing services. Secondly, it supports highly expressive policies representing any access structures. Thirdly, it offers a good solution to data confidentiality. As generating a secret key for a user happens only once but can be used to decrypt all the subsequent ciphertexts, CP-ABE reduces communication overhead [18]. Fourthly, it is collusion-resistant against misbehaving authorized users, which is achieved by associating a random number or polynomial with each attribute of a user’s secret key so that only the attributes with the same random value can be used for decryption, leading to preventing different legitimate users from colluding with each other [20]. Finally, it is possible to integrate CP-ABE with a proxy re-encryption technique in cloud in order to increase security by re-encrypting ciphertexts without disclosing the plaintexts to the cloud [18].

However, there are some weaknesses related to CP-ABE. These include the fact that CP-ABE only works fine when attributes are descriptive. In other words, temporal attributes are not well handled by CP-ABE. In addition, this technique is difficult to handle the attribute/user revocation problem [21] without trusting the cloud service provider that has already hosted the data, particularly in dynamic environments where users’ attributes can change over time. Trusting a cloud server raises another issue which is a collusion attack. This attack involves revoked users colluding with the cloud server to combine their information together to gain access to unauthorized data. Therefore, the adoption of CP-ABE requires additional refinements.

Based on the entities that a user can obtain authorization from, CP-ABE is classified into two different categories [22]. They are single authority CP-ABE, where all attributes are handled by a single authority, and multiauthority CP-ABE, in which different authorities manage the attributes in a distributed manner. However, in multiauthority systems, many complicated issues can be experienced when the CP-ABE systems are built. For example, to tie the work of all authorities together, some existing systems use either a central authority, which can cause a bottleneck problem and is contradictory to the distributed control principle [23], or coordination between the authorities, which increases communication and computational costs. In addition, each authority needs to be aware of each other, running the risk of collusion by combining their information to figure out unauthorized information. In addition, the revocation process is more complicated to manage in this type of system [14,23].

On the other hand, some issues can be encountered in any single authority systems. The first one is the key escrow problem that happens due to the ability of the authority to gain access to all users’ keys. This ability is obtained by the authority as it possesses the master key from which the users’ keys are derived. The second issue is the limited ability of any single authority system to handle a wide range of different attributes. Moreover, it represents a single-point bottleneck on security. Once an adversary compromises the system, the authority’s master key is easy to obtain.

To efficiently exploit the advantages of CP-ABE and avoid most of its drawbacks, we need to construct a new scheme of CP-ABE that mitigates the difficulties in the existing schemes and use sufficient, alternative solutions that dynamically change users’ privileges without entrusting information to a cloud server and build a collusion resistant system. The main issues that we are concerned about are

- Resolving the revocation problem.
- Covering a wide range of attributes needed by any system and eliminating a single point failure.
- Reducing the computation overhead.

### 3.2. The Revocation Problem

Revocation is a property to change the access rights of users when unexpected events occur, such as malicious behavior from a user or an expired service that a user had purchased [14]. There are two scenarios where the revocation can be conducted. The first one is called attribute revocation that happens when some of a user’s attributes are removed from the current set due to being degraded in the system. For example, degrading a manager of an organization to a normal employee role leads to losing some of its possessed attributes and hence access rights. The second scenario occurs when a user leaves the system; its access rights have to be revoked so that the user is no longer able to access the system and decrypt any stored data on it, which is called user revocation [24]. Based on these considerations, designing a mechanism to revoke the user’s certain access rights must be embedded in the system from the beginning. Otherwise, the whole system has to be rebuilt with the advent of each revocation event.

In attribute-based encryption schemes, the attribute revocation is a severe problem and very costly to apply for two reasons. The first one is the same attribute set may be associated with different users’ secret keys, causing significant computational overhead throughout the revocation process. This happens due to the need for updating all relevant keys for the non-revoked users and re-encrypting the related ciphertexts [14]. The second reason is most of the existing proposed attribute revocation methods are based on a fully trusted server, but this is an unrealistic assumption [24]. This is because the server could breach the trust and even be compromised, resulting in permitting unauthorized users to access the data stored in the cloud for gaining profits, e.g., when the cloud illegitimately permits a company to access the data of its competitors.

Therefore, the following crucial requirements are needed to handle the revocation problem.

- Permit instantaneous banning of a malicious user.
- Resist collusion attacks or invalidate the secret keys of the revoked users [14] (which means the cloud cannot collude with a revoked user to illegally obtain encrypted data).
- Minimize the computation overhead of the revocation process.
- Support forward security which means any newly published ciphertext cannot be decrypted by any revoked user with revoked attributes [25].

Although considerable research has been devoted to solving the revocation issue, most of the existing studies lack practicality, and the revocation process is considered as a major hindrance (Table 2 illustrates the main existing systems). The current strategies and assumptions utilized for the revocation are either considering that the server used is a trusted entity that can be assigned critical essential, auxiliary processes of access control or, in the worse-case scenario, assuming that the data owner and a private key generator (attribute authority) stay online all the time [24].

Some studies have been done to handle the revocation problem periodically [26,27]. Wan et al. [26] propose a hierarchical attribute set-based encryption scheme with user revocation. To cope with user revocation, they added an attribute expiration time to a user’s key. This time indicates the validity period of the user’s key. However, this causes serious vulnerabilities due to the uncontrolled period from the revocation time of a user to the expiration time of its key, as well as bringing an extra computational burden to the authority for frequent key updating and maintaining secure channels for all transactions. On the other hand, other schemes have also been proposed with instantaneous attribute revocation [14,24,28,29].

Several researchers have worked to build systems that resist collusion attacks [30,31]. These schemes [30,31] use a secret sharing scheme in order to prevent the server from decrypting the ciphertexts or illegitimately granting permissions to revoked users to access the data. In addition, they achieve dynamic, immediate attribute and user revocation without updating keys of non-revoked users. However, these schemes can only revoke a limited number of users. On the other hand, some schemes can revoke an unlimited number of users [19]. In Li et al.’s scheme [19], a unique identifier is associated with every user’s secret key, which, in turn, is used to construct the revocation information to be embedded in the ciphertext. However, the ciphertext size increases linearly with the number of revoked users, which has a negative impact on available storage capacities, particularly when the amount of data is large.

Moreover, applying the revocation process consumes a lot of computing resources. The attribute revocable system proposed in Yang and Jia’s scheme [28] needs to update all keys for the non-revoked users and re-encrypt the related ciphertext, which leads to low scalability and high computational overheads. Other recent studies have utilized a refereed delegation of computation models to alleviate the computation overhead of the identity-based encryption during the revocation process [32,33]. These schemes introduce an aided server, which is a Key Update Cloud Service Provider, to outsource most operations of the key generation related to the revocation process. For each user they assign a hybrid private key containing two types of components—identity and time components—which are combined together using the AND gate. The users need to periodically contact the Key Update Cloud Service Provider to update their time components according to a revocation list. However, this approach requires the server to be honest.

Moreover, some studies have developed an attribute revocation process using techniques based on issuing versions of users’ secret keys [24,29]. In Chen and Ma’s scheme [29], each user uses the old versions of its secret key to get the newest one, which leads to storing all versions of updated keys in the cloud to avoid a stateless receiver problem, which occurs when users lose their previous keys needed to compute their updated secret keys. However, keeping records of all the previous secret keys leads to a storage overhead. To overcome this problem, another mechanism has been proposed. Only the latest secret key needs to be held by its corresponding users in Yang et al.’s scheme [24]. Instead of updating all the non-revoked users’ secret keys and re-encrypting the ciphertexts, only the components in the secret keys and ciphertexts associated with the revoked attributes need to be updated. The workload of ciphertext update is delegated to a server. Although this system improves the efficiency of the attribute revocation mechanism and reduces the storage overhead, it requires the cloud server to be semi-trusted.

A dynamic user revocation scheme was proposed by Xu et al. [34]. In this scheme, the cloud server is in charge of re-encrypting ciphertext by using its assigned delegation key. However, this scheme does not handle the attribute revocation. So, a user will lose its access right of accessing data in the system, when it is put on the revocation list even if it still has other access attributes. However, some studies have enabled CP-ABE with proxy re-encryption that transforms a ciphertext of a message into another ciphertext of the same message by a semi-trusted proxy server using a re-encrypting key without any knowledge of the underlying plaintext [35] to achieve the attribute and user revocation [36]. In the scheme of Zu et al. [36], two master keys are generated by the authority. One is sent to the cloud server to deal with the revocation process, and the other is used to derive the secret keys of users. So, when a revocation event occurs, the non-revoked users’ access rights would not be affected. Although this scheme does not need to update keys in the case of attribute revocation, there is a need to re-encrypt the ciphertext.

In addition, some studies have been proposed to accelerate the revocation process by applying a mechanism to change just the affected part of data instead of the entire one [37]. In such a scheme, the data is split into a number of slices using the variant of a secret sharing scheme which is called All or Nothing, and then it is outsourced to the cloud. When a revocation process happens, only one slice needs to be retrieved by the data owner in order to re-encrypt and then re-upload it. However, the data owner must conduct the revocation process. To overcome the problem of the owner having to stay online all the time, the revocation process may not be executed immediately and also requires additional computation costs.

Recently, some works have been concerned about the attribute revocation issue. In Wang and Wang’s scheme [38], the authors proposed a system which addresses the problem of revoking users and attributes dynamically. The revocation process is executed by the cloud server, which re-encrypts the ciphertext according to the revocation list using the proxy re-encryption techniques and responds to the queries of the non-revoked users, as well as partially decrypts the ciphertext for them. Moreover, the cloud server has additional shares of the system attributes that are used for attribute revocation. In this way, revoking one attribute from some users’ privileges will not affect the access of other legitimate users. Although the system outsources heavy computational tasks to a cloud server (in particular, re-encryption and part of decryption operations), as well as addressing the problem of revoking users and attributes dynamically, the cloud is required to be semi-trusted. Therefore, the system does not resist against collusion attacks and partly grants the cloud server more control over data access. Moreover, the ciphertext size in this system increases linearly with the number of revoked users due to an additional ciphertext header and other components.

Furthermore, adding new attributes to an updated access policy is a critical mission which some of the existing systems do not manage. However, the work in Yuan’s system [39] addresses this problem. Although the access policy is enforced cryptographically, it can be changed dynamically without updating users’ secret keys. A dynamic policy update process is needed to transform an old linear secret sharing (LSS) matrix to an updated one corresponding to their relevant policies. When the two matrices are compared, the attributes changed by the access policy updating and the corresponding vectors in the matrix will be recognized to change only the ciphertext components associated with those updated attributes. The distribution of the re-encrypted ciphertext after updating the policy is similar to the distribution of the old ciphertext. However, many changes frequently occur in a set of ciphertext components and these changes are done by the data owner. This means an additional computational burden on the data owner. Moreover, the system re-randomizes the ciphertext before updating it. The re-randomization cost is similar to the cost of the whole ciphertext encryption process which leads to communication and computation overheads.

Moreover, some schemes have been built in a multiauthority cloud environment, where, the attribute revocation problem is a more complicated issue. Most of the existing revocation techniques are either not efficient or are based on a trusted server. Therefore it is not sufficient to apply them to multiauthority schemes [40]. The scheme of Abraham and Sriramya [22] does not require a server to be totally trusted, because the updating of keys is carried out by each attribute authority and not by the cloud server. However, in this revocable scheme, the burden of the revocation process is shifted to the authorities, which, in turn, are exposed to corruption due to periodically communicating to system users. On the other hand, an identity-based revocation technique used in a multiauthority system is introduced [14], which leads to distributing the computational overhead over a large number of users when they run the encryption and decryption algorithms. However, the computational burden for revocation has negative effects on the users.

In cloud storage systems, granting trust to a server that is curious about a user’s privacy or maintaining the data owner online all the time, is not an appropriate situation. In addition, the shortcomings of the existing schemes are (a) a lazy revocation process implying delay in revocation, (b) issuing new secret keys to the non-revoked users, (c) re-encrypting ciphertexts during user/attribute revocation, and (d) expanding the ciphertext size. All these criteria highlight the real need for building a system, which securely outsources expensive computations to a server without any leakage of private information so as to achieve privacy-preserving revocation. This will achieve two essential perquisites. The first one is to prevent the cloud from colluding with the revoked users or gaining any information about the plaintext and the other is to reduce the computation cost on the data owner.

## 4. The Types of the CP-ABE Scheme

In terms of distributed control in an untrusted cloud environment, and based on the way of granting authorization to users (i.e., depending on gaining secret keys by users from a single trusted entity or from a group of independent cooperative entities), the CP-ABE schemes can be classified into two categories that are described below.

### 4.1. The Single Authority Scheme

Most of the existing systems employ one entity to have the power of generating the decryption private keys for all system users. In such schemes, one attribute authority administrates all system attributes. This authority has the master secret key that is used to derive all users’ decryption secret keys. These keys are distributed to the system users via secret channels. The inherent issue of this type of schemes is the key escrow problem that occurs due to the ability of an attribute authority to recover any ciphertext using its master key. However, the security assumption that such systems is based on, is that the authority is fully trusted. On the other hand, crashing or corrupting this entity affects the availability of the whole system.

In addition to the key escrow issue, due to the lack of schemes that can efficiently address some issues of CP-ABE, such as revocation and collusion resistance, many researchers have been motivated to construct a practical CP-ABE system using a single authority scheme (Table 3 shows a summary of these studies). Notably, some approaches have been introduced to eliminate the computation cost [41] for lightweight devices. This type of study uses a CP-ABE scheme to offer constant sizes for both ciphertexts and secret keys. It uses one-way hash functions and an encryption algorithm to produce a ciphertext and a special polynomial function to generate a secret key with a key generation algorithm. However, it only supports the AND gate access structure. In addition, in this proposed scheme, the revocation problem is not taken into account.

Furthermore, some recent work has been proposed to alleviate the computations to be appropriate for resource-limited mobile devices [42,43,44]. In Fu et al.’s system [42], the system is introduced with a large attribute universe based access control, when the space and number of system attributes are flexible and not limited in the setup phase, outsources decryption to the cloud. This single-authority system uses the linear secret sharing (LSS) access structure. The works in Liu et al. and Li et al.’s schemes [43,44] use an online–offline technique to eliminate most computations. The online/offline CP-ABE scheme in [43] is proposed to mitigate the online encryption computation burden on an e-healthcare record (EHR) owner by splitting these computations into offline computations, which are performed before knowing the data and specifying the access policy, and a few online computations which are required to keep long-lasting battery life. In this scheme, the linear secret sharing scheme is used to encode the access policy. However, these systems [42,43,44] do not address the revocation problem.

In the scenario of encrypting medical records where a data owner (patient) ought to generate a secret key to system users, outsourcing the key generation is desirable. Therefore, some researchers have proposed a fully outsourced ABE scheme [45] that achieves outsourced key generation, encryption, and decryption. The system supports the LSS access structure. In terms of outsourcing the key generation and reducing the communication cost (e.g., battery consumption), the server will generate an intermediate secret key with only knowing the public key which can be downloaded later by the user after charging its mobile without draining the battery (i.e., the outsourcing operation is offline). To protect the master secret key and the private keys, the data owners hire two different servers to generate secret keys. However, the two servers colluding between each other means the whole system is under collusion attacks. Furthermore, the revocation problem is not considered in this system.

Moreover, outsourcing the heavy operations of CP-ABE and fog computing has also attracted a lot of research attention [46]. Fog computing is a paradigm extended from the cloud computing. Such a scheme [46] uses an access tree as an access structure. The system proposes an approach to outsource part of the encryption and decryption operations to fog nodes in order to minimize the computational burden on the data owners and system users, respectively. In addition, the system addresses the attribute change by updating the secret keys for all affected system users who share the updated attribute. However, sending the updated key to those users via a secure channel causes communication and computation overheads. Furthermore, the system assumes that the cloud service provider, fog nodes, and the attribute authority are fully trusted.

On the other hand, some studies have been carried out to reduce the computation cost of the ABE by using a pairing-free ABE system [47,48]. In Karati and Amin’s system [47], the system also eliminates the transmission overhead on the secret channel by sending a large part of a secret key on a public channel to the users, while sending only the blinding factor via a secret channel. However, using the pairing instead is more reliable and secure. In addition, this scheme does not use the LSS to distribute the attributes in the users’ secret keys, which is a more expressive access structure than the threshold scheme that this scheme uses. Moreover, the revocation problem is not taken into account by the authors.

In Hong and Sun’s scheme [48], besides reducing the complexity of ABE by using pairing-free ABE, invalidating the leaked keys of non-revoked users and the revoked keys is considered. When a key of a non-revoked user is accidently leaked or revoked upon attribute revocation, a key insulation technique is utilized to divide the system lifetime into several periods. At each period of time, only one part of the secret key can be updated by the authority which computes the updating components and sends them to the authorized users. The authority uses a random number for each period of time. The system supports the tree access structure. However, the system does not provide an instant key invalidation operation. Alternatively, the revocation happens periodically. Furthermore, a heavy computation cost is imposed on data owners thanks to re-encrypting a plaintext at each period of time.

Furthermore, some work has been done to not only outsource the decryption operation to a cloud server, but also further verify that the outsourced decryption carried out by the cloud is correct [49]. The verification technique transforms a ciphertext using some processes with a blinded secret key. Then, before computing the plaintext, a user compares each transformed ciphertext component with the corresponding, original ciphertext component to retrieve the blinded value. In this case, the outsourced decryption is verified. The tree access structure is used in this system. However, the system uses a big size of ciphertext as well as incurring a heavy computation cost. In addition, the revocation problem facing any access control system is not addressed.

As a result of the importance of protecting health information, which may be revealed by access policies, some studies have been proposed to hide an access policy in CP-ABE schemes [50]. This system [50] uses a large attribute universe and partially hides the access policy. The system handles any expressive access policies represented as a linear secret sharing scheme (LSSS). However, some additional computational operations are added before the decryption phase for testing whether a user’s attributes satisfy the access policy, imposing more burdens on a user. Furthermore, the scheme does not consider the revocation problem.

In addition, recent studies have been proposed to achieve more security by hiding the access policy [51,52]. In Han et al.’s scheme [52], the authors present a CP-ABE scheme that provides two features. The first is to hide the attribute values from the attribute authority. This happens by utilizing a 1-out-of-n oblivious transfer technique that can send attributes in a fuzzy selection manner to the authority; in this case, the authority can generate the secret key without knowing the attribute value. The second is to protect the type of attribute in the access policy that is embedded in the ciphertext using an attribute bloom filter approach to check whether an attribute belongs to the hidden access policy without revealing it. The LSSS access structure is supported in this system. However, more computational operations are incurred by a data owner and users. These operations increase linearly with the complexity increase of the access structure and the number of the users’ attributes. Furthermore, in terms of communication overhead, more information needs to be sent to the attribute authority. In addition, the revocation problem is not managed.

Although most of the existing systems can hide access structures and support restricted access structures with a composite-order group, of which the order is a product of two large primes, the scheme of Cui et al. [51] introduces a mechanism to partially hide access structures by enabling the expressive LSSS access structure in a prime-order group, which is a cyclic group with a prime number order. Pairing performance in a scheme with a composite order group is ~50 times lower than the same pairing in the prime order group [53]. In general, each attribute consists of a name and a value. In this scheme [51], the attributes’ values in the access policy are hidden by the data owner due to their sensitivity. The authors use a randomness splitting mechanism to protect the values of the attributes by hiding them in the ciphertext. However, the revocation problem is not dealt with. Furthermore, expensive operations are needed to compute the ciphertext and each user’s private key as well as increasing the size of the ciphertext.

In summary, it is essential to consider the problems that single authority schemes create. These include (1) the diversity of attributes that are hard to manage by only one authority, (2) the key escrow problem that occurs when the single authority is not totally trustworthy and has the ability to gain access to all users’ keys, and (3) the security failure that creates a serious problem when the authority is compromised by an adversary that gains the system’s master key. A multiauthority scheme is suitable for resolving these weaknesses.

### 4.2. The Multiauthority Attribute Based Access Control System

To tackle the single authority schemes’ problems, an effective way is to minimize the trust level of the single authority, strengthen the privacy of user data, and enhance the system security and performance by replacing the single authority with multiple ones for disjoint attribute management, which becomes much harder for an adversary to compromise. Therefore, in this section, the type of scheme that allows securely storing data on a public cloud storage system and employs multiple authorities to manage sets of attributes, is presented.

A critical challenge of current multiauthority access control systems (also all single authority schemes) is the inefficiency of the key generation process. This issue occurs in the single authority systems when one authority manages all attributes in a system and issues secret keys for all system users. Therefore, compromising or crashing this authority makes the whole system unavailable. The same issue happens in multiple authorities schemes, when each authority in the system administrates a disjoint attribute set (i.e., each authority administrates a different set of attributes), which presents a performance bottleneck. To mitigate the effects of this issue, all attribute sets ought to be managed by all system attribute authorities individually.

Although many recent multiauthority CP-ABE schemes have been proposed [54,55], some limitations are still not considered. The existing multiauthority access control systems can be classified into three categories with their limitations summarized below.

- The first type of scheme (e.g., a scheme by Han et al. [56]) contains many authorities that have to work together, resulting in a high communication cost and lack of scalability since it is hard for authorities to join or leave freely. Furthermore, these authorities might collude with each other and combine their information to gain unauthorized data about the users.
- The second type needs a central authority to tie the work of all authorities together, and to be involved in issuing users’ secret keys besides having the master key (e.g., the work by Liu et al. [57]). The drawbacks of this type of scheme are that the concept seems contradictory to distributed control and it incurs low performance and a security bottleneck.
- Decentralized systems are the third type of the multiauthority access control system, which remove any central authority and employ independent attribute authorities, where the systems are scalable (e.g., the system proposed by Ruj et al. [58]). For this type of system, user revocation is hard to address, which incurs a heavy computation cost.

The first multiauthority access control system was proposed by Chase et al. [59]. This system uses a central authority as an active entity, which generates users’ secret keys, co-operates with the system attribute authorities that manage disjoint attribute sets and distribute the secret keys. The problem with this system is that the central authority has the master key that can be used to decrypt all ciphertexts. This means that the central authority represents a performance and security bottleneck. The system also does not address the revocation problem.

Yang et al. [60] proposed a decentralized access control model by using multi authorities with a semi-active central authority which is only in charge of initializing the system. In their system, part of the decryption operation is outsourced to a cloud server to mitigate the burden of decryption on a user. Moreover, the system supports the revocation process. However, in the revocation phase, heavy computation is put on attribute authorities (AAs) for computing an update key for each non-revoked user. Since the attributes change frequently, this approach becomes a performance killer and not practical in cloud access control systems. In addition, the attribute set in this system is divided into various disjoint subsets where each one is driven by one authority. Once an authority is compromised, the adversary can gain the corresponding private keys of its attributes, which, in turn, affects the performance of the whole system.

Another multiauthority scheme [61] has been proposed to advance the system in [60] by jointly managing a system attribute set. In this work, a verifiable threshold multiauthority access control model with a semi-active central authority is introduced using a secret sharing approach to generate a shared master key among multiple authorities, where all the attribute authorities collaborate with each other to create the key. In this scheme, user’s secret keys can be generated by contacting a threshold number of attribute authorities. However, this system does not address the revocation problem. In addition, some communication and computation are needed among the authorities to exchange their key shares and reconstruct the master key. Furthermore, a heavy computation workload is placed on users.

Moreover, some researchers have claimed that they have proposed a revocable threshold multiauthority access control system with the management of joint attribute sets [62] to advance the system in [61]. However, the theoretical model presented in this work uses an access tree as an access structure, unlike the scheme in [61], which uses a LSSS as an access structure. Moreover, the theoretical model shows that the system is performed as a single authority access control system, not as what the authors have claimed. Furthermore, the system does not address the attribute revocation problem. In addition, the experimental results are vague. The same issue occurs with another study [63], which is again not as what its authors have claimed.

Administrating joint attribute sets is advocated in Xue et al.’s system [64]. This system adopts the technique in Li et al.’s work [61] and employs a framework to eliminate communication costs by efficiently assigning a part of the secret key generation task to the central authority. Since this assigning operation is based on receiving intermediate keys from ***t*** attribute authorities, where the keys are associated with attributes, this operation does not affect negatively on solving the single-point performance bottleneck problem with the other systems. However, the system does not address the revocation problem and assumes that the central authority is fully trusted and has the master key, meaning that the compromised central authority with one corrupted attribute authority can break the system security.

Although CP-ABE schemes give data owners more control over their data, a decentralized system with multiple, uncoordinated authorities has been proposed to increase data owners’ control over the data by giving them more privileges to restrict access to a fraction of the data [65]. In this scheme, even if a user’s attribute set fulfills a data owner’s access policy, the user can decrypt a fraction of a related ciphertext according to how many fractions are specified by the data owner. The data owner encrypts its whole data once using one policy and different symmetric keys. The approach utilized is chunk based encryption, which divides data into several chunks and a different symmetric key is used to encrypt each chunk. This scheme uses LSSS as an access structure. However, although the scheme improves the encryption process, it makes the key generation process more complicated. In addition, it does not manage the revocation problem.

Recently, some studies have been carried out to devise a decentralized multiauthority system with no central authority and without any interactions among the authorities involved [66], where the attribute sets are disjoint. The authors proposed an approach to hide the access policy and resolve the revocation problem. However, upon each revocation event, expensive computational operations are needed. These include updating the secret keys for all non-revoked users after generating an updated key (containing the new version of the revoked attributes) by the authorities and re-encrypting the components of the ciphertext already stored on the cloud server and associated with the revoked attributes. Furthermore, heavy computations are put on the data owner due to its heavy responsibilities. These include the data owner’s responsibilities for encrypting the ciphertext, hiding the access policy by replacing each attribute in the access policy by a value of pairing between the hash value of that attribute and the public key of the authority that manages this attribute, re-encrypting the ciphertext, and sending it to the cloud server.

Another recent work is proposed by Li et al. [67]. It uses a decentralized multiauthority scheme with accountability to trace the misbehaving users who leak their decryption keys. In the system, each attribute authority deals with a disjoint attribute set. Although there is no central authority in the system, there are some interactions among the authorities to share a secret function. In addition, the system hides the attribute information in the ciphertext. However, the system uses an AND gate access policy in an inflexible way, and also managing the accountability leads to an increased ciphertext size because part of the ciphertext that deals with an access policy, is specified to include authorized users’ identities, with ‘*’ being used if no specific identity is required. Furthermore, the number of authorities is defined in the system initialization phase, which means the system is not scalable afterwards. Moreover, in this system, the most critical revocation issue is not addressed.

Some other recent studies have claimed to solve the problem of key escrow, prevent the key-abuse attack and increase the level of single authority trust by proposing an accountable authority [68,69] instead of using a multiauthority scheme. The work of Ning et al. [68] proposes two accountable, revocable systems that manage the accountability, traceability, and privilege revocation of malicious users. However, in these two systems, the researchers use two techniques for revocation. The first one uses a revocation list and embeds it in ciphertexts, leading to an increase in ciphertext size. The second approach implements periodical key updating that comes with an additional cost of communications and computations by issuing an updated key for non-revoked users. Moreover, it is not an effective solution to use an accountable authority as an alternative to multiple authorities due to the lack of administrating a wide range of attributes. The work uses a composite order group, which needs complicated processes and makes the system less efficient than a prime order group.

In addition, some researchers proposed a multiauthority scheme with a hidden structure attribute-based encryption [70]. They use a tree access structure and disjoint attribute sets. In this system, a central authority plays a main role in creating the system master key sent to all attribute authorities. This concept works similarly to a single authority approach, which lacks decentralization and represents a security bottleneck. Moreover, the system inefficiently addresses the revocation problem. After each revocation process, the central authority has to update the revocation list, reissue a new master secret key and send it to all the authorities in the system in a secure manner. These authorities then regenerate new secret keys for all non-revoked users.

To sum up, a multiauthority CP-ABE scheme is an appropriate solution to addressing security and privacy issues as well as enhancing the performance in the cloud environment. However, the current work has several limitations with notable ones listed in Table 4, including inefficient revocation, high communication and computation costs, and inefficient key generation. These challenges highlight an urgent need to propose a multiauthority scheme that can not only securely outsource expensive computations to cloud without revealing private information but also efficiently control user access privileges. Outsourcing computations to the cloud reduces the computation costs on data owners and users while allowing user access privileges to be efficiently elevated or revoked according to a policy update process.

## 5. Research Challenges and Future Directions

For further work, a number of desirable features need to be provided in any effective multiauthority access control systems. Such features are described as follows.

- Management of joint attribute sets to efficiently generate users’ secret keys. The reason for this is that if each authority manages a different set of attributes, compromising or crashing an authority causes unavailability of the whole system, which presents a performance bottleneck.
- Dealing with the single point of security failure that all single authority systems and some multiauthority schemes suffer from. Furthermore, no one entity in a system should have full control of all the information.
- The handling of the revocation issues should enable the dynamicity and flexibility of customizing and managing users’ access privileges as well as protecting the system from collusion attacks.
- Outsourcing the expensive encryption and decryption operations to cloud servers should alleviate the computational burden on data owners and system users without revealing unauthorized information.
- Achieving accountability. In an untrusted environment, one of the potential research directions is to make an access control system accountable for protection against key exposure. Such exposure could occur when the secret decryption key of an authorized user is leaked. Since this key is valid, the decryption of the corresponding ciphertext is possible. Therefore, an effective mechanism is needed to protect the system from this threat.
- Reducing communication overhead. Investigating an approach to reduce communications among system entities would be one of the key aspects to improve the efficiency of that system.
- Hiding access policies. For sensitive policies, one of substantial future research tasks is to explore techniques to enforce the access policies in a ciphertext form for the protection of their private information during policy deployment.
- Using dynamic attributes. Another core property that the CP-ABE technique needs to be extended with is dynamic attributes (e.g., location or time). It is an open challenge that would improve the dynamicity of a system by adding attributes to users’ secret keys to restrict cloud data access in response to attribute changes and to enable a dynamic adaptation scheme.

## 6. Conclusions

In this paper, the benefits, requirements, challenges, and weaknesses of outsourcing and sharing data in the cloud environment are presented. We have focused on the most critical issues that the existing single and multiple authorities CP-ABE schemes have not efficiently addressed, such as the revocation problem. A number of current revocation techniques have been critically analyzed. Moreover, the access structures used by the surveyed studies have been identified. Apart from the existing survey papers, this paper has critically assessed the relevant work from various aspects. For further work, we have highlighted the urgent need to propose a scheme that can not only securely outsource expensive computations to cloud but also administrate a wide range of attributes and efficiently control user access privileges.

## Figures and Tables

**Table 1:** Summary of the proposed ABE schemes.

Scheme	Description	Revocation	Access Policy
Bethencourt et al. [9]	The first CP-ABE scheme using a tree access structure	Lack of Revocation	Less expressive
Waters [10]	The first fully expressive CP-ABE scheme using a linear secret sharing access structure	Lack of Revocation	Full expressive
Wang et al. [17]	The first hierarchical ABE scheme with a disjunctive normal form (DNF) policy	Addressing revocation	Not expressive

**Table 2:** Classifying main existing systems based on the revocation type.

Scheme	Description	Revocation Type	The Problem
[26,27]	Add an attribute expiration time to a user’s key	User revocation	Periodically
[30,31]	Resist collusion attacks	Attribute and user revocation	Limited number
[19]	Revoke an unlimited number of users	User revocation	The ciphertext size increases linearly with the number of revoked users
[28]	Consume a lot of computing resources	Attribute revocation	High computation overhead
[32,33]	Alleviate the computation overhead	Periodic attribute revocation	Collusion attack
[24,29]	Issue versions of users’ secret keys	Attribute revocation	Collusion attack
[34]	Dynamic revocation	User revocation	No attribute revocation
[35]	Enable CP-ABE with proxy re-encryption	Attribute revocation	High computation overhead
[36]	Use two master keys	Attribute and user revocation	Collusion attack
[37]	Accelerate the revocation	User revocation	Computational burden on a data owner
[38]	Dynamic revocation	Attribute and user revocation	Collusion attack
[39]	Updated access policy	Attribute revocation	Computational burden on a data owner

**Table 3:** Summary of main existing systems and their limitations.

Scheme	Description	Access Structure	The Problem
Odelu et al. [41]	Eliminate the computation cost for lightweight devices	AND gate	Lack of revocation
Fu et al. [42]	Provide large attribute universe based access control	LSSS	Lack of revocation
Liu et al. and Li et al. [43,44]	Offer online–offline techniques to eliminate most computations	LSSS	Lack of revocation
Zhang et al. [45]	Propose a fully outsourced ABE scheme	LSSS	Lack of revocation
Zhan et al. [46]	Outsource the heavy operations of CP-ABE to fog computing	Access Tree	Inefficient revocation
Karati et al. [47]	Reduce the computation cost of ABE by using pairing-free ABE	Threshold	Lack of revocation
Hong and Sun [48]	Reduce the computation cost of ABE by using pairing-free ABE	Access Tree	Periodical revocation
Kumar et al. [49]	Outsource and verify the decryption operation	Access Tree	Lack of revocation
Zhang et al. [50]	Hide an access policy in CP-ABE schemes	LSSS	Lack of revocation
Cui et al. and Han et al. [51,52]	Hide an access policy in CP-ABE schemes	LSSS	Lack of revocation

**Table 4:** Summary of main existing systems and their limitations.

Scheme	Description	The Problem	Attribute Set
Han et al. [56]	The authorities have to work with each other	High communication cost	Disjoint
Liu et al. [57]	Using an active central authority to administrate attributes	Security bottleneck	Disjoint
Lin et al. [71]	Decentralized threshold authorities work together without a central authority	Lack of revocation	Disjoint
Ruj et al. [58]	Decentralized system without a central authority	Lack of revocation	Disjoint
Li et al. [72]	The central authority is not involved in generating secret keys	Support AND access structure which is not expressive	Disjoint
